# Molecular Analysis of High-Grade Serous Ovarian Carcinoma Exhibiting Low-Grade Serous Carcinoma and Serous Borderline Tumor

**DOI:** 10.3390/cimb46090555

**Published:** 2024-08-25

**Authors:** Kosuke Kanno, Kentaro Nakayama, Sultana Razia, Sohel Hasibul Islam, Zahan Umme Farzana, Shahataj Begum Sonia, Hiroki Sasamori, Hitomi Yamashita, Tomoka Ishibashi, Masako Ishikawa, Kayo Imamura, Noriyoshi Ishikawa, Satoru Kyo

**Affiliations:** 1 Department of Obstetrics and Gynecology, Shimane University Faculty of Medicine, Izumo 693-8501, Japan; kanno39@med.shimane-u.ac.jp (K.K.); hasibulsohel1167@gmail.com (S.H.I.); farzanashormi99@gmail.com (Z.U.F.); sbsonia1995@gmail.com (S.B.S.); sasamori@med.shimane-u.ac.jp (H.S.); memedasudasu1103@gmail.com (H.Y.); m-ishi@med.shimane-u.ac.jp (M.I.); aphrodite.41.41@icloud.com (K.I.); 2 Department of Obstetrics and Gynecology, Nagoya City University East Medical Center, Nagoya 464-8547, Japan; tomoka@med.nagoya-cu.ac.jp; 3 Department of Legal Medicine, Shimane University Faculty of Medicine, Izumo 693-8501, Japan; raeedah-med@yahoo.com; 4 Department of Pathology, Shonan Fujisawa Tokushukai Hospital, Fujisawa 251-0041, Japan; noriyoshi.ishikawa@tokushukai.jp

**Keywords:** ovarian cancer, high-grade serous carcinoma, low-grade serous carcinoma, serous borderline tumor, *MDM2*

## Abstract

Ovarian cancer is classified as type 1 or 2, representing low- and high-grade serous carcinoma (LGSC and HGSC), respectively. LGSC arises from serous borderline tumor (SBT) in a stepwise manner, while HGSC develops from serous tubal intraepithelial carcinoma (STIC). Rarely, HGSC develops from SBT and LGSC. Herein, we describe the case of a patient with HGSC who presented with SBT and LGSC, and in whom we analyzed the molecular mechanisms of carcinogenesis. We performed primary debulking surgery, resulting in a suboptimal simple total hysterectomy and bilateral salpingo-oophorectomy due to strong adhesions. The diagnosis was stage IIIC HGSC, pT3bcN0cM0, but the tumor contained SBT and LGSC lesions. After surgery, TC (Paclitaxel + Carbopratin) + bevacizumab therapy was administered as adjuvant chemotherapy followed by bevacizumab as maintenance therapy. The tumor was chemo-resistant and caused ileus, and bevacizumab therapy was conducted only twice. Next-Generation Sequencing revealed *KRAS* (p.G12V) and *NF2* (p.W184*) mutations in all lesions. Interestingly, the TP53 mutation was not detected in every lesion, and immunohistochemistry showed those lesions with wild-type p53. *MDM2* was amplified in the HGSC lesions. DNA methylation analysis did not show differentially methylated regions. This case suggests that SBT and LGSC may transform into HGSC via p53 dysfunction due to *MDM2* amplification.

## 1. Introduction

Ovarian cancer is the leading cause of death among Japanese females diagnosed with gynecological cancers [1]. According to the World Health Organization (WHO) Classification of tumors of the ovary 2020, ovarian cancers are classified into high-grade serous carcinoma (HGSC; 34.4%), clear cell adenocarcinoma (23.7%), endometrioid adenocarcinoma (17.5%), mucinous adenocarcinoma (8.3%), low-grade serous carcinoma (LGSC; 1.6%), and malignant Brenner tumor (0.2%) [2,3]. Those subtypes are further classified into type I and type II according to their molecular, histopathological, and clinical features. Type I tumors comprise LGSC, mucinous carcinoma, endometrioid carcinoma, clear cell carcinoma, and malignant Brenner tumor, while Type II tumors include HGSC, undifferentiated carcinoma, and malignant mixed mesodermal tumor [4].

LGSC arises from serous borderline tumors (SBTs) in a stepwise manner, while HGSC develops from serous tubal intraepithelial carcinoma (STIC) [5,6,7]. In terms of genetic alterations, *BRAF* and *KRAS* mutations are detected in >60% of LGSC [4], while the *TP53* mutation is detected in over 95% of HGSC [8] and other mutations occur in less than 5% of the HGSC [6]. Both the *KRAS* mutation and *BRAF* mutation activate the RAS/RAF/mitogen-activated protein kinase (MAPK) kinase (MEK)/MAPK signaling pathway and promote cell proliferation and inhibit cell apoptosis [9]. Thus, *KRAS* and *BRAF* mutations are found even in up to 67% of SBTs, whose mutations are regarded as non-sufficient for carcinogenesis [5]. The key mechanism of transformation from SBTs to LGSC is still unknown. Gene expression is regulated by not only genetic mechanisms but also epigenetic mechanisms such as DNA methylation. The methylation of CpG islands alters the activity of DNA transcription factor binding sites and results in a loss of expression of the gene [10,11]. Focusing on serous neoplasms, Shih et al. reported that SBT and LGSC are hypermethylated compared to HGSC [11]. These pathologically, genetically, and epigenetically different subtypes can co-exist. Moreover, SBT and LGSC can develop into HGSC; however, this is rare [12].

Here, we present an HGSC case in a 75-year-old female presenting with SBT and LGSC lesions. We further assess the molecular mechanisms underlying its carcinogenesis.

## 2. Materials and Methods

### 2.1. Patient Information

The patient was a 75-year-old female who complained of abdominal distension. She visited a local clinic where computed tomography (CT) and ascites cytology were performed. The CT scan revealed a left ovarian tumor, while ascites cytology showed adenocarcinoma. She was then referred to Shimane University Hospital. Subsequent contrast-enhanced CT and magnetic resonance imaging (MRI) showed a 12 cm left ovarian tumor, omental cake, and >2 cm peritoneal dissemination. There was no indication of lymph node involvement or distant metastasis. A primary debulking surgery was performed comprising a total hysterectomy and bilateral salpingo-oophorectomy; thus, a diagnostic laparoscopy was not a standard therapy at that time. Due to significant intraabdominal adhesions, it was not possible to perform an omentectomy, a resection of disseminated tissue, or lymph node dissection. The patient was ultimately diagnosed with high-grade serous ovarian carcinoma Stage IIIC (FIGO 2014), pT3bcN0cM0(UICC 8th).

Following surgery, TC therapy (Paclitaxel and Carboplatin) was administered as adjuvant chemotherapy. However, Olaparib + Bevacizumab or Niraparib maintenance therapy had not yet been covered by insurance. No BRCA mutation was detected via BRCAnalysis^®^ (Myriad genetics, Salt Lake City, UT, USA); thus, Bevacizumab was added from the 2nd cycle. After three cycles of this chemotherapy, the tumor status was stable disease (SD); thus, we did not conduct additional surgery and continued with the chemotherapy. After six cycles, Bevacizumab was administered as maintenance therapy. However, maintenance therapy was only administered twice as the tumor was resistant to Bevacizumab and developed, causing ileus. The patient died nine months after the primary surgery.

### 2.2. Immunohistochemistry

Immunohistochemistry was conducted with the following materials: rabbit polyclonal anti-Estrogen receptor alpha (ab75635; Abcam, Cambridge, UK), rabbit monoclonal anti-progesterone receptor (ab32085; Abcam, Cambridge, UK), p53 (DO-7; ROCHE, Basel, Switzerland), Ki67 (30-9; ROCHE, Basel, Switzerland), CK7 (OV-TL 12/30; DAKO, Nowy Sącz, Poland), and WT1 (6F-H2; ROCHE, Basel, Switzerland)). Staining procedures followed the manufacturer’s instructions.

### 2.3. DNA Extraction and Next-Generation Sequencing (NGS)

Each lesion was reviewed and marked under hematoxylin and eosin (HE) staining by a skilled gynecologic pathologist (N.I.). Total DNA was extracted following a previously described method [13,14] with slight modifications. Briefly, we placed each lesion on a membrane slide and stained it with hematoxylin. We then dissected each slide manually to purify each lesion; the carcinoma/stroma ratio of SBT, LGSC, and HGSC was 70%, 80%, and 90%, respectively. Then, each dissected sample was digested with proteinase K overnight and DNA extraction was conducted with the QIAmp DNA FFPE Tissue Kit (Qiagen, Valencia, CA, USA). Each DNA concentration of HGSC, LGSC, and SBT was 1067.4 ng/μL, 309.2 ng/μL, and 323.2 ng/μL, respectively. Extracted DNA was processed with the Illumina Miseq sequencing platform (Illumina, San Diego, CA, USA) for 160 cancer-related genes (Supplementary Table S1). The results were analyzed in the Genomejack bioinformatics pipeline (Mitsubishi Space Software, Tokyo, Japan) for annotation and curation. The NGS system we used was an internal clinical sequencing named the “PleSSision Panel” test, which analyzes exome regions. We obtained the somatic gene alterations such as single nucleotide variations (SNVs), insertions or deletions, and copy number (CV) variations (CNVs). The CN was calculated as a mean value of all reads covering the target gene and was compared with the average of the control sample (peripheral blood). We previously described the PleSSision test [15].

### 2.4. DNA Methylation Analysis

The remaining DNA samples were analyzed with the Infinium MethylationEPIC Kit and GenomeStudio Software V2011.1 (Illumina, San Diego, CA, USA) to assess the methylation status. The DNA methylation score (β) ranges from 0 (unmethylated) to 1 (methylated); Δβ represents the difference between β of each lesion and normal tissue. If Δβ is positive, the gene is more methylated, whereas if Δβ is negative, the gene is more unmethylated. We defined hypermethylated genes as those with Δβ ≥ 0.2 and hypomethylated genes as those with Δβ ≤ −0.2.

## 3. Results

### 3.1. Pathological Analysis

**Figure 1:**
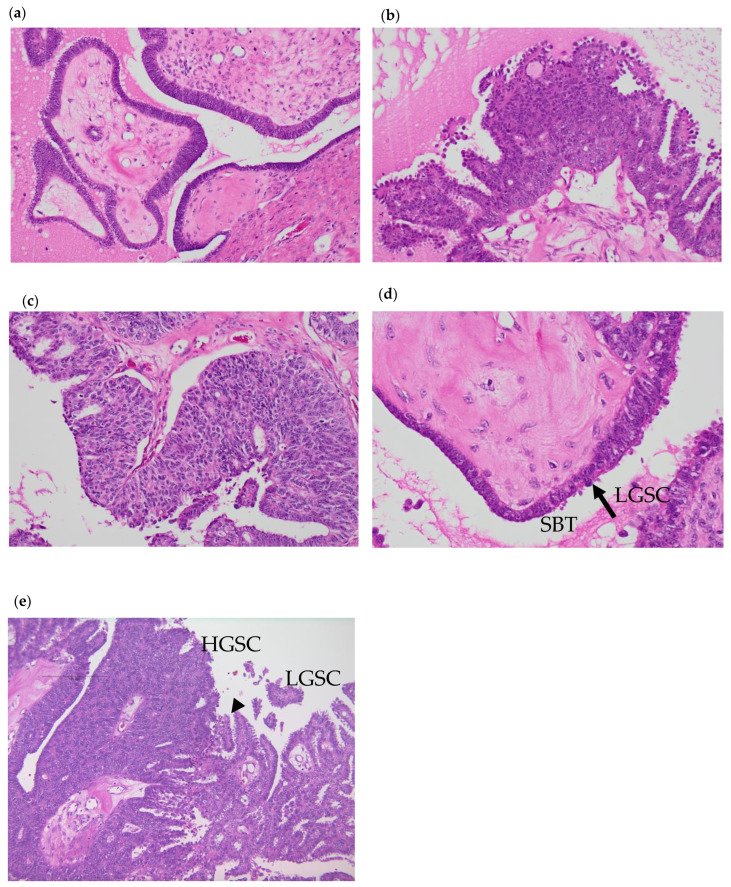
Hematoxylin and eosin (HE) staining of each lesion. (**a**) The SBT lesion at 200× *g* magnification. (**b**) The LGSC lesion at 200× *g* magnification. (**c**) HGSC lesion at 200× *g* magnification. (**d**) The arrow indicates junction of SBT and LGSC. SBT is contiguous with LGSC at 400× *g* magnification. (**e**) The arrow head indicates junction of LGSC and HGSC. LGSC is contiguous with HGSC at 100× *g* magnification.

A specialized pathologist diagnosed the tumor. Histological SBT and LGSC lesions were identified in H&E staining in this case (Figure 1). Major invasive lesions and severely complexed papillary structures were observed, comprising highly atypical cells and corresponding to HGSC lesions. Adjacent to the HGSC lesion, mild to moderate atypical cells formed an invasive papillary structure, which corresponds with LGSC lesions. Mild atypical papillary structures without invasion were also mixed with the lesions. Accordingly, we concluded that HGSC lesions, LGSC lesions, and SBT lesions coexisted. Meanwhile, p53 immunohistochemistry (IHC) revealed wild-type expression in all lesions. The results of IHC are summarized in Table 1 and Supplementary Figure S1. Ki67 expression was high in HGSC, while there was a low expression in LGSC and little expression in SBT. WT1 and CK7 were all positive in every lesion. Estrogen receptor (ER) was weakly positive in SBT and HGSC, whereas it was positive in LGSC. Progesterone receptor (PR) was positive in every lesion, but only partially in SBT and HGSC.

All lesions showed wild-type p53 expression. Ki67 expression was highest in HGSC, while it was lowest in SBT. WT1 and CK7 were all positive in every lesion. ER was weakly positive in SBT and HGSC, whereas it was positive in LGSC. PR was positive in every lesion, but only weakly and partially in SBT and HGSC.

### 3.2. Genomic Analysis

**Figure 2:**
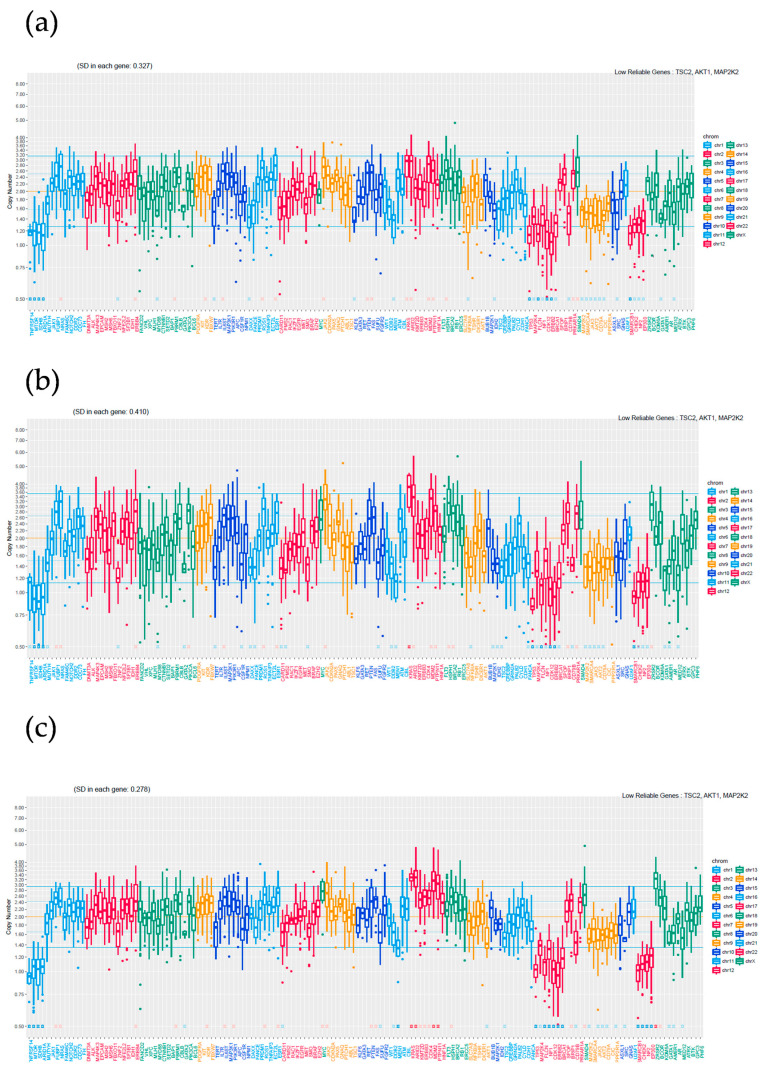
Copy number alterations. (**a**) SBT; (**b**) LGSC; (**c**) HGSC. The difference between MDM2 is greater in HGSC compared to SBT and LGSC.

The mean depths of SBT, LGSC, and HGSC were 620.1, 589.3, and 719.2, respectively. Tumor cellularities approximated based on variant allele frequencies of SBT, LGSC, and HGSC were 70%, 80%, and 90%, respectively. All detected mutations are shown in Table 2. *KRAS* p.G12V (NM_004985.5:c.35G>T, NP_004976.2:p.Gly12Va, HGVS nomenclature) and *NF2* p.W184* (ENST00000338641.4:c.551G>A, ENSP00000344666.4:p.Trp184Ter, HGVS nomenclature) were the only mutations detected in the three lesions. Moreover, the variant allele frequency (VAF) of these mutations increased sequentially in the order SBT < LGSC < HGSC. Copy number (CN) alterations are presented in Table 3 and Figure 2. The same genes were amplified or lost in the three lesions; however, additional alterations were detected in HGSC compared with LGSC. More specifically, *MDM2* was amplified, while *EP300*, *MEN1*, and *NF1* CNs were lower in HGSC compared with LGSC and SBT (Table 3, Supplementary Table S2).

### 3.3. Epigenetic Analysis

**Figure 3:**
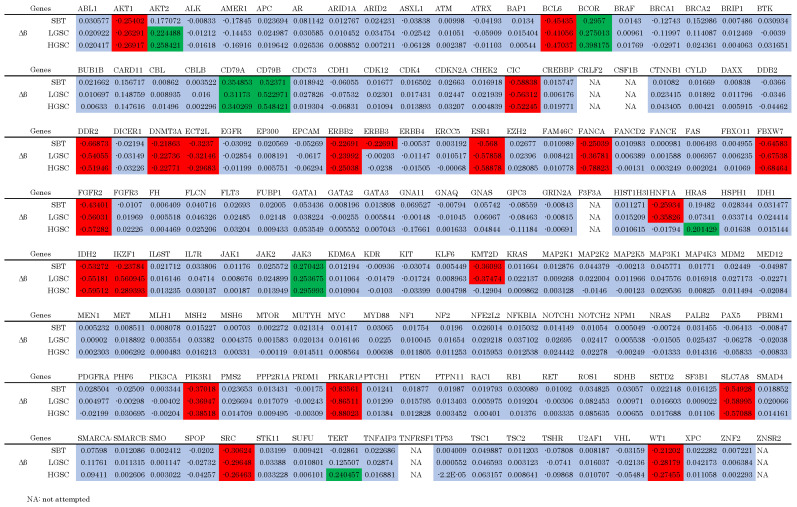
Epigenetic analysis of cancer-related genes. Green-colored cells indicate hypermethylated genes. Red-colored cells indicate hypomethylated genes. Blue-colored cells indicate equally methylated genes. No oncogenes are hypomethylated and no tumor suppressor genes are hypermethylated specifically in HGSC.

We hypothesized that the HGSC lesion was systemically less methylated than the other lesions, while TSGs were more methylated. A total of 46,562 probes were examined; 2995 (6.432%), 2958 (6.352%), and 3551 (7.626%) methylated probes were detected in the SBT, LGSC, and HGSC lesions, respectively. Subsequently, we assessed the cancer-related genes identified via NGS (Figure 3). No oncogenes (Ogs) or tumor suppressor genes (TGSs) were specifically hypomethylated or hypermethylated in the HGSC lesion, respectively.

## 4. Discussion

In the current case, HGSC, LGSC, and SBT lesions were pathologically adjacent. Moreover, NGS detected the same mutations among the lesions, with the same genes amplified or lost across those lesions; these alterations were most apparent in the HGSC lesion. Those results suggest that the HGSC, LGSC, and SBT lesions not only comprised a coincidental tumor but also had the same origin.

IHC and NGS also revealed wild-type *TP53* expression in each lesion. Given that most classical HGSCs have *TP53* mutations [8], it was postulated that the HGSC lesion in this case was not a classical tumor. Moreover, NGS detected the *KRAS* mutation in the HGSC lesion, which occurs in 67% of LGSC cases [4]. Hence, the current HGSC lesion likely contained LGSC aspects. Collectively, these findings suggest that HGSC arose from SBT/LGSC.

IHC was positive for WT1, CK7, and ER. WT1 is useful in distinguishing HGSCs and LGSCs from clear cell carcinomas (CCCs) and mucinous carcinomas (MCs); WT1 is diffusely expressed in most HGSCs and LGSCs, whereas it is negative in most CCCs and MCs [16]. Positive CK7 is usually used to distinguish metastasis from primary lower gastrointestinal cancer [17]. PR was negative in LGSC, but only <50% of traditional LGSCs showed PR expression. Ki67 expression was high in HGSC, but low in LGSC and SBT. Those results are consistent with previous reports [18,19,20,21,22]. Intact p53 was the only difference between our HGSC lesion and other HGSCs. This may be the characteristics of this mixed-type serous carcinoma.

Despite our hypothesis, methylation rates were similar within each lesion. Hence, epigenetic mechanisms other than OG hypomethylation and TSG hypermethylation may have played an important role in this SBT/LGSC transformation.

There are a few studies that analyzed those mixed-type serous carcinomas. For example, Zarei et al. analyzed ovarian serous carcinoma mixed with HGSC and LGSC [12] and found that 66% of those mixed types had no mutated genes that are commonly found in solid tumors. The *TP53* mutation was detected only in 22.2%. Other mutations were one *NRAS* mutation and one *BRAF* mutation. Murali et al. reported five cases of mixed-type serous carcinomas and found that only two cases had *TP53* mutations while three cases had *KRAS* or *NRAS* mutations [23]. Vang et al. analyzed TCGA study samples and reviewed 14 cases with wild-type *TP53* sequences. Only one case was HGSC with LGSC, and it lacked *TP53* mutations, a large number of mutations, and frequent CN alterations [24]. Chui et al. analyzed 1025 serous carcinoma cases who had SBT beforehand, and found that among three cases of HGSC cases, two had *KRAS* mutations [25].

Focusing on TP53-wild-type HGSC, Chui et al. also analyzed 987 HGSC cases and found *TP53*-wild-type in only 2.5% of them [26]. Among their 25 *TP53*-wild-type HGSCs, 24% (6 cases) had *KRAS*, *BRAF,* or *NRAS* mutations. These studies suggest that RAS/MAPK signaling activation or TP53-wild-type is characteristic of mixed-type serous carcinoma. Ahmed et al. analyzed 123 high-grade serous carcinomas and found four cases of *TP53*-wild-type HGSC [27]. Among them, *MDM2* or *MDM4* copy number gain was observed in three cases. This *MDM2* amplification was found in other *TP53*-wild-type HGSC cases [26]. *MDM2* suppresses p53 expression by concealing the *TP53* activation domain, activating p53 ubiquitination, and exporting p53 to the cytoplasm [28,29]. In the present study, CNV analysis indicated a slight *TP53* CN loss in the LGSC and HGSC, and *MDM2* amplification in the HGSC lesion. Additionally, although no *TP53* mutations were detected, *MDM2* may have weakened p53 functions, representing a possible key transformation mechanism.

This study has certain limitations. First, this is a single case analysis. Hence, additional analyses, including similar cases, may reveal other key mechanisms. Given that serous carcinomas that present as SBT or LGSC and HGSC comprise only 2–3.4% of all ovarian cancer [12,30], multi-institutional joint research may be necessary. Second, we were unable to analyze disseminated lesions or relapsed lesions. Inoue et al. reported a case who was diagnosed with HGSC when she underwent partial omentectomy as the primary surgery, whereas the SBT component was found in the second surgery after six cycles of combined chemotherapy of docetaxel and carboplatin [31]. The tumors showed different chemo-sensitivity; the HGSC component showed chemo-sensitivity, while the SBT component showed chemo-refractory. As such, a future research question includes determining whether disseminated/relapsed lesions are HGSC, LGSC, or mixed. Due to tumor heterogeneity, the primary tumor and disseminated lesions might exhibit different results. Finally, our epigenetic analysis only comprised DNA methylation. Other epigenetic mechanisms such as mRNA methylation may play important roles in the transformation of LGSC to HGSC.

## 5. Conclusions

Herein, we presented an unclassical ovarian HGSC mixed with SBT and LGSC. Genomic and epigenomic analysis suggested that the key mechanisms underlying the transformation of SBT and LGSC to HGSC may be p53 dysfunction owing to MDM2 amplification.

## Figures and Tables

**Table 1:** IHC profile of 3 lesions of the present case.

	SBT	LGSC	HGSC
p53	wild-type	wild-type	wild-type
Ki67	low	low	high
WT1	positive	positive	positive
CK7	positive	positive	positive
ER	weak positive	positive	weak positive
PR	partially positive	negative	partially positive

ER: estrogen receptor, PR: progesterone receptor.

**Table 2:** Detected mutations in the three lesions.

Mutation		VAF	
	SBT	LGSC	HGSC
*KRAS* p.G12V	49.2%	56.5%	60.3%
*NF2* p.W184*	59.0%	66.6%	81.4%

VAF: variant allele frequency. *KRAS* p.G12V and *NF2* p.W184* were detected in every lesion.

**Table 3:** Copy number alterations.

Chromosome	Gene		Estimated CN	
		SBT	LGSC	HGSC
chr11	*MEN1*	1.11	0.95	1.27
chr12	*MDM2*	3.2	3.66	3.3
chr17	*NF1*	1.27	1.37	1.25
chr22	*EP300*	1.09	0.95	1.14

CN: copy number. Standard deviations (SDs) of SBT, LGSC, and HGSC were 0.327, 0.410, and 0.278, respectively.

## Data Availability

The data presented in this study are available on request from the corresponding author (K.N.).

## Supplementary Materials

The following supporting information can be downloaded at https://www.mdpi.com/article/10.3390/cimb46090555/s1. Figure S1: IHC of each lesion; Table S1: Analyzed cancer-related genes; Table S2: All copy number alterations.
