# Supplementary material for: Molecular Analysis of High-Grade Serous Ovarian Carcinoma Exhibiting Low-Grade Serous Carcinoma and Serous Borderline Tumor
Source: Curr Issues Mol Biol. 2024 Aug 25;46(9):9376–85. doi: 10.3390/cimb46090555 (PMC11430742; doi:10.3390/cimb46090555)
Supplement: Supplementary file 1 [file cimb-46-00555-s001.zip › cimb-3154051-supplementary.pdf]

Molecular Analysis of High-grade Serous Ovarian Carcinoma Exhibiting Low-grade Serous Carcinoma and Serous Borderline Tumor

Supplementary Materials

a-1.)

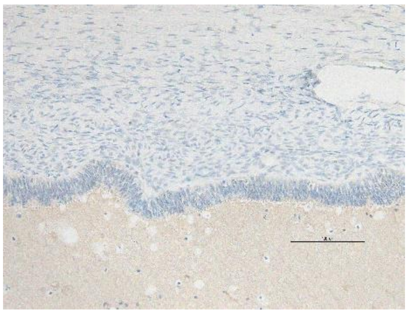

a-2.)

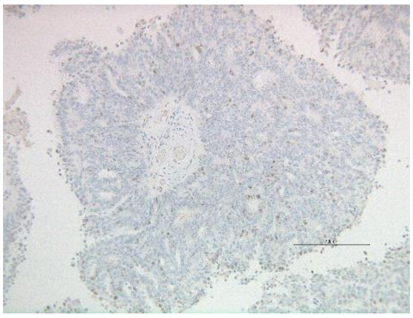

a-3.)

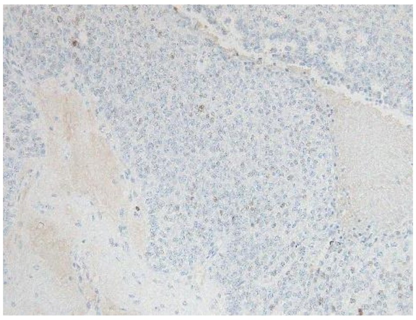

b-1.)

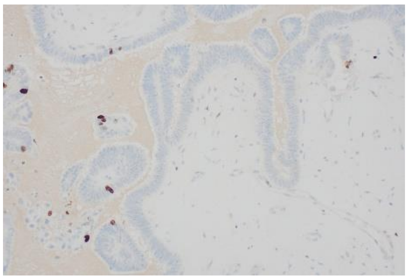

b-2.)

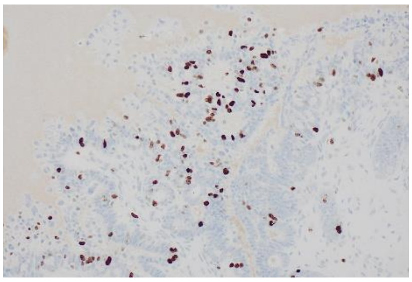

b-3.)

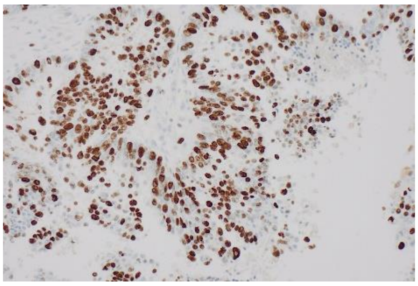

c-1.)

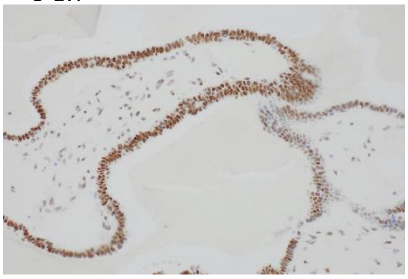

c-2.)

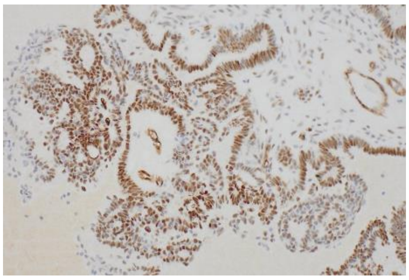

c-3.)

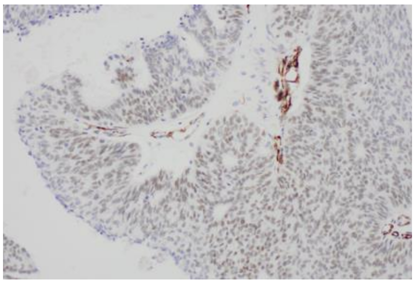

d-1.)

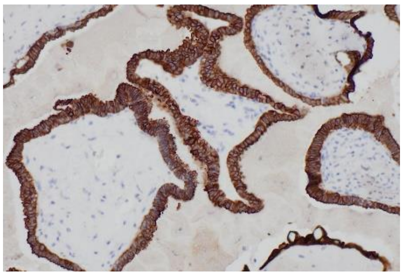

d-2.)

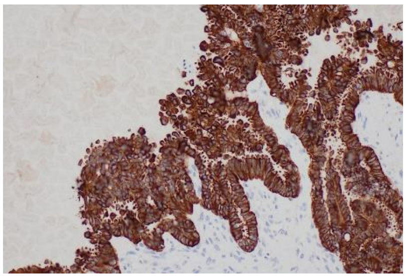

d-3.)

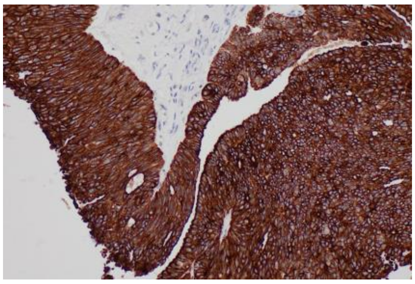

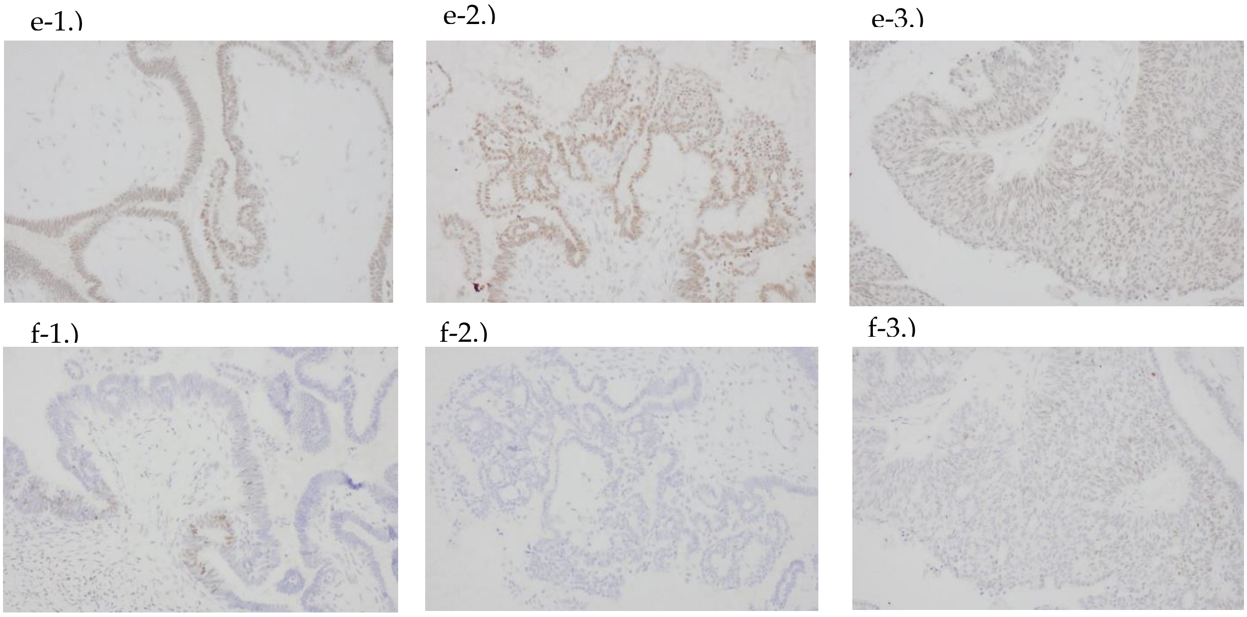

Supplementary Figure S1. IHC of each lesion.

Every 1), 2), 3) are SBT, LGSC, HGSC lesions, respectively. a.) p53 IHC showed wild type pattern in all lesions. b.) Ki-67 IHC. HGSC showed high expression, while LGSC showed less and SBT showed little expression. c.) WT-1 IHC showed positive in all lesions. d.) CK7 IHC showed positive in all lesions e.) ER IHC showed positive in LGSC and HGSC, while weak positive in SBT. f.) PR IHC showed partially positive in SBT and HGSC, and negative in LGSC.

Supplementary Table S1. Analyzed cancer related genes.

ABL1	DDR2	IDH2	PDGFRA
AKT1	DICER1	IKZF1	PHF6
AKT2	DNMT3A	IL6ST	PIK3CA
ALK	ECT2L	IL7R	PIK3R1
AMER1	EGFR	JAK1	PMS2
APC	EP300	JAK2	PPP2R1A
AR	EPCAM	JAK3	PRDM1
ARID1A	ERBB2	KDM6A	PRKAR1A
ARID2	ERBB3	KDR	PTCH1
ASXL1	ERBB4	KIT	PTEN
ATM	ERCC5	KLF6	PTPN11
ATRX	ESR1	KMT2D	RAC1
BAP1	EZH2	KRAS	RB1
BCL6	FAM46C	MAP2K1	RET
BCOR	FANCA	MAP2K2	ROS1
BRAF	FANCD2	MAP2K5	SDHB
BRCA1	FANCE	MAP3K1	SETD2
BRCA2	FAS	MAP4K3	SF3B1
BRIP1	FBXO11	MDM2	SLC7A8
BTK	FBXW7	MED12	SMAD4
BUB1B	FGFR2	MEN1	SMARCA4
CARD11	FGFR3	MET	SMARCB1
CBL	FH	MLH1	SMO
CBLB	FLCN	MSH2	SPOP
CD79A	FLT3	MSH6	SRC
CD79B	FUBP1	MTOR	STK11
CDC73	GATA1	MUTYH	SUFU
CDH1	GATA2	MYC	TERT
CDK12	GATA3	MYD88	TNFAIP3
CDK4	GNA11	NF1	TNFRSF14
CDKN2A	GNAQ	NF2	TP53
CHEK2	GNAS	NFE2L2	TSC1
CIC	GPC3	NFKBIA	TSC2
CREBBP	GRIN2A	NOTCH1	TSHR
CRLF2	F3F3A	NOTCH2	U2AF1
CSF1B	HIST1H3B	NPM1	VHL
CTNNB1	HNF1A	NRAS	WT1
CYLD	HRAS	PALB2	XPC
DAXX	HSPH1	PAX5	ZNF2
DDB2	IDH1	PBRM1	ZNSR2

Supplementary Table S2. All copy number alterations

Chromosome	Gene	estimated CN		
		SBT	LGSC	HGSC
**chr1**	TNFRSF14	0.85	0.93	0.82
**chr1**	MTOR	0.86	0.64	0.96
**chr1**	SDHB	0.82	0.62	0.92
**chr1**	ARID1A	0.76	0.66	0.96
**chr1**	MUTYH	1.59	1.32	1.83
**chr1**	JAK1	2.32	1.98	2.18
**chr1**	FUBP1	2.64	3.01	2.6
**chr1**	NRAS	3.09	3.51	2.5
**chr1**	FAM46C	1.92	1.62	1.99
**chr1**	NOTCH2	2.6	2.22	2.35
**chr1**	DDR2	2.28	2.06	2.14
**chr1**	CDC73	2.4	2.8	2.27
**chr1**	FH	2.39	2.43	2.25
**chr2**	DNMT3A	1.69	1.42	1.72
**chr2**	ALK	1.9	1.59	1.87
**chr2**	MAP4K3	2.62	2.8	2.37
**chr2**	EPCAM	2.32	2.48	2.14
**chr2**	MSH2	2.22	2.26	2.19
**chr2**	MSH6	1.95	1.65	2
**chr2**	FBXO11	2.29	2.62	2.21
**chr2**	ZNF2	1.3	1.01	1.63
**chr2**	NFE2L2	2.22	2.22	2.19
**chr2**	SF3B1	2.48	2.69	2.38
**chr2**	IDH1	2.27	1.99	2.02
**chr2**	ERBB4	2.83	3.03	2.48
**chr3**	FANCD2	2.1	1.86	2.22
**chr3**	VHL	1.85	1.7	1.97
**chr3**	XPC	1.99	1.67	1.97
**chr3**	MLH1	2	1.8	2.16
**chr3**	MYD88	1.38	1.33	1.76
**chr3**	CTNNB1	2.16	2.12	2.21
**chr3**	SETD2	2.41	2.19	2.31

**chr3**	BAP1	1.59	1.52	1.84
**chr3**	PBRM1	2.8	2.57	2.52
**chr3**	CBLB	2.57	2.72	2.4
**chr3**	GATA2	1.49	1.19	1.56
**chr3**	PIK3CA	2.5	3.06	2.47
**chr3**	BCL6	2	1.75	2.04
**chr4**	PDGFRA	2.22	1.86	2.2
**chr4**	KIT	2.59	2.43	2.42
**chr4**	KDR	2.77	2.49	2.53
**chr4**	FBXW7	2.47	2.74	2.43
**chr5**	TERT	1.34	1.23	1.72
**chr5**	IL7R	2.07	2.03	2.23
**chr5**	IL6ST	2.85	2.86	2.63
**chr5**	MAP3K1	2.67	2.68	2.32
**chr5**	PIK3R1	2.6	2.73	2.55
**chr5**	APC	2.35	2.17	2.29
**chr5**	CSF1R	1.65	1.3	1.68
**chr5**	NPM1	1.96	2.14	2.07
**chr6**	DAXX	1.19	1.23	2.08
**chr6**	FANCE	1.53	1.23	1.72
**chr6**	PRDM1	2.28	2.08	2.25
**chr6**	ROS1	3.01	3.13	2.72
**chr6**	TNFAIP3	2.37	2	2.37
**chr6**	ECT2L	2.48	2.15	2.29
**chr6**	ESR1	3.12	2.96	2.79
**chr7**	CARD11	1.45	1.19	1.61
**chr7**	PMS2	1.5	1.18	1.83
**chr7**	RAC1	1.77	1.68	1.91
**chr7**	IKZF1	2.22	1.82	1.96
**chr7**	EGFR	2.08	1.74	2.01
**chr7**	MET	2.39	2.2	2.28
**chr7**	SMO	1.56	1.29	1.71
**chr7**	BRAF	2.3	2.42	2.15
**chr7**	EZH2	2.22	2.22	2.3
**chr8**	MYC	1.87	2.68	2.85

**chr9**	JAK2	3.07	3.61	2.78
**chr9**	CDKN2A	2.65	2.43	2.16
**chr9**	PAX5	2.31	1.96	2.02
**chr9**	GNAQ	2.67	2.59	2.39
**chr9**	PTCH1	2.18	1.79	2.12
**chr9**	ABL1	1.87	1.74	1.99
**chr9**	TSC1	2.1	1.84	2.05
**chr10**	KLF6	1.1	1.43	1.77
**chr10**	GATA3	1.82	1.84	2.11
**chr10**	RET	1.79	1.68	1.91
**chr10**	PTEN	2.77	2.71	2.48
**chr10**	FAS	2.79	2.77	2.56
**chr10**	SUFU	1.71	1.34	1.61
**chr10**	FGFR2	2.29	1.79	2.34
**chr11**	WT1	2.18	1.62	1.97
**chr11**	DDB2	1.5	1.31	1.61
**chr11**	MEN1	1.11	0.95	1.27
**chr11**	ATM	2.53	2.71	2.32
**chr11**	CBL	2.08	1.89	2.23
**chr12**	KRAS	3.37	4.32	3.44
**chr12**	ARID2	3.36	3.73	3.44
**chr12**	KMT2D	2.13	2.15	2.54
**chr12**	ERBB3	2.09	2.09	2.65
**chr12**	CDK4	2.06	2.23	2.59
**chr12**	MDM2	3.2	3.66	3.3
**chr12**	PTPN11	2.87	3.05	3.16
**chr12**	HNF1A	2.25	2.11	2.43
**chr13**	FLT3	2.34	2.06	2.19
**chr13**	HSPH1	2.88	3.42	2.65
**chr13**	BRCA2	2.51	2.94	2.47
**chr13**	RB1	2.24	2.58	2.19
**chr13**	ERCC5	2.58	2.33	2.22
**chr14**	SLC7A8	1.97	1.58	1.88
**chr14**	NFKBIA	1.25	1.24	1.72
**chr14**	TSHR	2.04	1.82	2.01

**chr14**	DICER1	2.31	2.25	2.16
**chr14**	AKT1	1.56	1.5	1.36
**chr15**	BUB1B	2.44	2.33	2.26
**chr15**	MAP2K1	1.8	1.3	1.83
**chr15**	IDH2	1.38	1.31	1.81
**chr16**	TSC2	1.43	1.24	1.47
**chr16**	CREBBP	1.75	1.4	1.86
**chr16**	GRIN2A	1.76	1.53	1.80
**chr16**	PALB2	1.96	1.7	2.05
**chr16**	CYLD	2.48	2.52	2.35
**chr16**	CDH1	1.95	1.44	1.91
**chr16**	FANCA	1.54	1.32	1.68
**chr17**	TP53	0.77	0.55	0.92
**chr17**	MAP2K4	1.21	1.15	1.32
**chr17**	FLCN	0.79	0.87	1.03
**chr17**	NF1	1.27	1.37	1.25
**chr17**	CDK12	0.84	0.77	0.92
**chr17**	ERBB2	0.63	0.68	0.84
**chr17**	BRCA1	1.04	0.98	1.09*
**chr17**	SPOP	2.34	2.26	2.15
**chr17**	BRIP1	2.70	2.99	2.51
**chr17**	CD79B	1.29	1.27	1.38
**chr17**	PRKAR1A	2.74	2.94	2.46
**chr18**	SMAD4	2.79	2.67	2.72
**chr19**	MAP2K2	1.39	1.24	1.45
**chr19**	SMARCA4	1.32	1.24	1.68
**chr19**	JAK3	1.28	1.07	1.43
**chr19**	AKT2	1.24	1.34	1.55
**chr19**	CD79A	1.00	1.24	1.5
**chr19**	CIC	1.24	1.44	1.64
**chr19**	PPP2R1A	1.59	1.41	1.53
**chr20**	ASXL1	1.70	1.48	1.79
**chr20**	SRC	1.31	1.43	1.47
**chr20**	GNAS	1.98	1.84	2.14
**chr21**	U2AF1	2.24	2.12	2.15

**chr22**	SMARCB1	0.8	0.69	0.91
**chr22**	CHEK2	1.01	0.94	1.00*
**chr22**	NF2	1.00	0.94	1.03
**chr22**	EP300	1.09	0.95	1.14
**chrX**	ZRSR2	2.41	3.34	3.38
**chrX**	BCOR	1.72	2.05	2.62
**chrX**	KDM6A	2.24	2.56	2.26
**chrX**	GATA1	1.15	1.16	1.38
**chrX**	AMER1	1.51	1.25	1.59
**chrX**	AR	1.94	1.64	1.95
**chrX**	MED12	1.34	1.06	1.48
**chrX**	ATRX	2.18	2.31	2.2
**chrX**	BTK	1.91	1.64	1.9
**chrX**	GPC3	2.18	2.09	2.10
**chrX**	PHF6	2.30	2.66	2.27
